# Quantitative Analysis of the Synergy of Doping and Nanostructuring of Oxide Photocatalysts

**DOI:** 10.3390/ma17143460

**Published:** 2024-07-12

**Authors:** Nicola Seriani, Paola Delcompare-Rodriguez, Dhanshree Pandey, Abhishek Kumar Adak, Vikram Mahamiya, Carlos Pinilla, Hala J. El-Khozondar

**Affiliations:** 1 The Abdus Salam International Centre for Theoretical Physics, Strada Costiera 11, 34151 Trieste, Italyaadak@ictp.it (A.K.A.);; 2 Istituto Officina dei Materiali, Consiglio Nazionale delle Ricerche (CNR-IOM), Via Bonomea 265, 34136 Trieste, Italy; 3 Departamento de Fisica y Geociencias, Universidad del Norte, Km 5, Via Puerto Colombia, Barranquilla 080020, Colombia; 4 Electrical Engineering and Smart Systems Department, Faculty of Engineering, Islamic University of Gaza, Gaza P.O. Box 108, Palestine

**Keywords:** metal oxides, characterization, electrochemical interface, Poisson–Boltzmann, hematite, doping, energy conversion

## Abstract

In this paper, the effect of doping and nanostructuring on the electrostatic potential across the electrochemical interface between a transition metal oxide and a water electrolyte is investigated by means of the Poisson–Boltzmann model. For spherical nanoparticles and nanorods, compact expressions for the limiting potentials at which the space charge layer includes the whole semiconductor are reported. We provide a quantitative analysis of the distribution of the potential drop between the solid and the liquid and show that the relative importance changes with doping. It is usually assumed that high doping improves charge dynamics in the semiconductor but reduces the width of the space charge layer. However, nanostructuring counterbalances the latter negative effect; we show quantitatively that in highly doped nanoparticles the space charge layer can occupy a similar volume fraction as in low-doped microparticles. Moreover, as shown by some recent experiments, under conditions of high doping the electric fields in the Helmholtz layer can be as high as 100 mV/Å, comparable to electric fields inducing freezing in water. This work provides a systematic quantitative framework for understanding the effects of doping and nanostructuring on electrochemical interfaces, and suggests that it is necessary to better characterize the interface at the atomistic level.

## 1. Introduction

In photoelectrocatalysis, the interface between the semiconducting photoelectrode and the electrolyte plays a central role; it is the place where excited charges and reactant molecules meet, and is where the actual chemical reaction takes place. As such, a full understanding of the structure of the interface and its dynamics under reaction conditions is desirable. Unfortunately, this is quite difficult because of the complexity of the system and reactions. The semiconductor is often doped and nanostructured, and the electrolyte often consists of water with soluted ions. An electrostatic potential is applied through the interface, and illumination induces a further redistribution of charges. This poses challenges to experimental characterisation techniques. Moreover, different aspects of the system are related to very different length and time scales; alignment of water molecules a few Ångstroms from the surface depends on the electric fields present there; however, these can often only be understood by considering the electrostatics of the whole interface, spanning up to hundreds of micrometers, or even of the whole device.

Much of today’s understanding remains based on continuum models that were developed decades ago [1,2,3,4], and provide a simplified picture of the system; analysis of the experimental data often relies on models that assume full depletion in the space charge layer, a planar interface, constant dielectric behaviour, and independence from atomic details. Although many of the shortcomings of these models are recognised [5,6,7], this insight often remains at the qualitative level. Therefore, it is highly desirable to devise simple analytic models that overcome some of the common assumptions in order to provide easier insight into the role of the different variables and can be used for systematic comparisons of experimental situations. For the case of relevance here, one important issue is that the capacitance of the Helmholtz layer becomes more important when the semiconductor is heavily doped. This is already known to the point that its values are sometimes extracted from electrochemical impedance spectroscopy data [8]. Nonetheless, the consequences are still often only discussed qualitatively. In addition, while nanostructuring is recognised as an important factor that can modify interface behaviour [6], it is rarely included in quantitative analysis, meaning that a systematic understanding of its effects is still missing. In the case of hematite, experiments on nanorods have been analysed while taking into account modified geometries [9,10]. It should be noted that analytic expressions are highly desirable, as they are easy to handle and provide conceptual insights into the role of the different properties. For this reason, we focus on a simple analytic treatment of the spherical and cylindrical cases. In this work, we study the interface in a continuum model, including the space charge layer, the Helmholtz layer, and the diffuse Gouy–Chapman layer. The model considers the presence of doping for both a spherical nanoparticle and a nanorod, and we compare the results to those for the planar interface [11]. The results are focused on two systems of particular interest, namely, that of a photoelectrode constituted by doped hematite (α−Fe_2_O_3_), and that in which the photoelectrode is constituted by titania (TiO_2_). Hematite is considered a promising photoanode for water splitting [12,13], and has been extensively studied both experimentally [7,14,15,16,17,18,19,20,21,22,23,24,25,26,27] and theoretically [28,29,30,31,32,33,34,35] in recent years, while titania was historically the first oxide to display photocatalytic water splitting [36,37] and remains the reference material in the field [38,39,40,41].

These two cases are highly interesting because the electrostatic fields that regulate charge dynamics both towards the interface and at the interface itself play a crucial role in ensuring that holes reach the interface in high concentrations and that the reaction kinetics are favourable [24,27,38]. We quantitatively analyse the interplay of doping and nanostructuring. Low doping ensures that the space charge layer is quite extended, favouring charge collection over recombination, while the potential that a nanostructure can sustain before being completely depleted is quite small. On the other hand, high doping reduces the size of the space charge layer, while nanostructuring is decisive in ensuring that the space charge layer represents a large volume fraction of the semiconductor. Finally, we show that at high doping electric fields in the Helmholtz layer can be as high as those that are known to cause freezing in pure water [42]. This raises questions about the dynamics of water at the interface in the presence of high doping and high applied potential.

In the following sections, we first discuss the analytic model, then present the results for the electrochemical interface between the oxide and a water electrolyte. Finally, we close the article with our conclusions.

## 2. Computational Methods

### 2.1. Poisson–Boltzmann Equations in the Spherical Case

**Figure 1:**
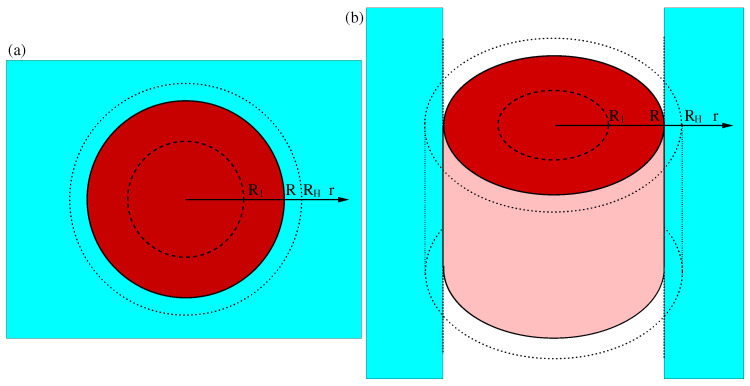
Geometry of (**a**) the spherical model of a nanoparticle and (**b**) the cylindrical model of an infinite nanorod. The dark red region is a cross-section of the solid, the pink region represents the outer surface of the solid, and the light blue region represents the electrolyte. $R_{1}$ is the inner boundary of the space charge layer, R is the radius of the nanostructure, and $R_{H}$ is the radius that encloses the Helmholtz layer.

The model describes the space charge layer in the depletion approximation, the Helmholtz layer, and the diffuse Gouy–Chapman layer. The assumptions are analogous to those employed in [11] for the planar interface. The Poisson–Boltzmann model assumes that the interface consists of three regions: the space charge layer in the semiconductor, the Helmholtz layer in the liquid in direct contact with the solid, and then the diffuse or Gouy–Chapman layer, also in the liquid. The space charge layer has a thickness that depends on the applied potential, and contains a fixed and uniform density of donors; the corresponding electrons are completely removed from the space charge layer (depletion approximation), meaning that the charge of the space charge layer is uniquely determined by its width and by the donor density. The Helmholtz layer is the thin region between the surface of the semiconductor and the closest point at which dissolved ions can come to the surface; it behaves like a capacitor with a given dielectric constant. Finally, the diffuse or Gouy–Chapman layer extends into the liquid, in principle to infinity, and the concentration of ions is determined self-consistently by Boltzmann statistics and the electrostatic potential. In the planar case, the equations have an exact analytic solution [11,43,44]. For the spherical case, an approximate analytic solution is available, with an exact expression for the space charge and Helmholtz layers and a robust analytic approximation for the diffuse Gouy–Chapman layer [45]. Before introducing a similar robust approximation below for the cylindrical case, we first discuss the spherical case. In this case, the semiconductor is a sphere; as shown in Figure 1a, the semiconductor has a radius R; the space charge layer is located in the region between a radius R_1_ and R (0 ≤ $R_{1}\leq$ R), the Helmholtz layer is between R and $R_{H}$, and beyond $R_{H}$ is the diffuse Gouy–Chapman layer. The boundary conditions are $\phi_{SC}\left(R_{1}\right)=0$, $\frac{\partial \phi_{SC}}{\partial r}\left(R_{1}\right)=0$, $lim_{r->\infty }\phi_{el}\left(r\right)=\phi_{el}\left(+\infty \right)$. The first condition is a choice of the zero of the potential, the second is a continuity condition for the electric field, which is strictly zero for r ≤ $R_{1}$, and the third amounts to fixing the total potential drop across the whole interface.

Here, we summarize the results; a detailed derivation is in the Supplementary Materials. The potential drop across the electrochemical interface is

(1) $$V_{app}-V_{fb}=\Delta \phi_{total}=\Delta \phi_{sc}+\Delta \phi_{H}+\Delta \phi_{el},$$

where $V_{app}$ is the applied potential, $V_{fb}$ is the flatband potential, and $\Delta \phi_{sc}$, $\Delta \phi_{H}$, and $\Delta \phi_{el}$ are the potential drops across the space charge layer, Helmholtz layer, and Gouy–Chapman layer, respectively. As in [11], we express all quantities as a function of $\Delta \phi_{SC}$. We also use the quantities

(2) $$A_{0}=\frac{6\epsilon_{0}\epsilon_{SC}}{eN_{D}}$$

and

(3) $$B=e\sqrt{\frac{2c_{0}}{kT\epsilon_{0}\epsilon_{el}}}.$$

Given a certain radius $R_{1}$ for the inner border of the space charge layer, the potential drop is

(4) $$\Delta \phi_{SC}=\frac{eN_{D}}{6\epsilon_{0}\epsilon_{SC}}\frac{1}{R}\left(R^{3}-3R_{1}^{2}R+2R_{1}^{3}\right).$$

Conversely, for a given potential drop in the space charge layer, the inner border of the space charge layer is located in

(5) $$R_{1}=R\left\{\frac{1}{2}+cos\left(\frac{1}{3}arccos\left(-1+2\frac{A_{0}}{R^{2}}\Delta \phi_{SC}\right)-\frac{2\pi }{3}\right)\right\}.$$

The other two contributions to the potential drop are

(6) $$\Delta \phi_{H}=\frac{eN_{D}}{3\epsilon_{0}\epsilon_{H}}\left(R^{3}-R_{1}^{3}\right)\left(\frac{1}{R}-\frac{1}{R_{H}}\right),$$

(7) $$\begin{array}{c} \phi_{el}\left(R_{H}\right)-\phi_{el}\left(+\infty \right)=\frac{2kT}{e}ln\{\frac{e}{2kT}\frac{\epsilon_{SC}}{\epsilon_{el}}\frac{d\phi_{SC}}{dr}\left(R\right)\frac{R^{2}}{R_{H}}\frac{1}{1-BR_{H}}\\ +\sqrt{1+{\left(\frac{e}{2kT}\frac{\epsilon_{SC}}{\epsilon_{el}}\frac{d\phi_{SC}}{dr}\left(R\right)\frac{R^{2}}{R_{H}}\frac{1}{1-BR_{H}}\right)}^{2}}\}, \end{array}$$

where

(8) $$\frac{\partial \phi_{SC}}{\partial r}\left(R\right)=-\frac{eN_{D}}{3\epsilon_{0}\epsilon_{SC}}\left(R-\frac{R_{1}^{3}}{R^{2}}\right).$$

These are the equations that define the potential drop across the electrochemical interface. As discussed in the Supplementary Materials. they reduce to the planar case in the limit of infinite radius. These expressions are valid in the depletion approximation for $0\leq \Delta \phi_{SC}\leq \Delta \phi_{SC}^{limit-sph}$, with

(9) $$\Delta \phi_{SC}^{limit-sph}=\frac{eN_{D}R^{2}}{6\epsilon_{0}\epsilon_{SC}}=\frac{R^{2}}{A}.$$

At this limiting value of the potential drop, the space charge layer includes the whole semiconductor sphere, and cannot be extended further. This last formula can be used to check whether the limit has been reached during an experiment.

### 2.2. Poisson–Boltzmann Equations in the Cylindrical Case

The cylindrical case is very interesting in view of the existence of nanostructures with cylindrical symmetry, such as nanotubes and nanorods. We use a similar notation to the previous section: the semiconductor is a cylinder of radius R, and the space charge layer is located in the region between a radius $R_{1}$ and R (0 ≤ $R_{1}\leq$ R); R is a geometric feature of the nanostructure, while $R_{1}$ depends on the applied bias. The general boundary conditions are $\phi_{SC}\left(R_{1}\right)=0$, $\frac{\partial \phi_{SC}}{\partial r}\left(R_{1}\right)=0$, $lim_{r->\infty }\phi_{el}\left(r\right)=\phi_{el}\left(+\infty \right)$; the first condition fixes the zero of the potential, the second condition forces the electric field to go to zero continuously at $R_{1}$, and the third condition fixes the potential difference between the center of the cylinder and the region far from the cylinder. The solutions are as follows,

(10) $$\Delta \phi_{SC}=\phi_{SC}\left(R_{1}\right)-\phi_{SC}\left(R\right)=\frac{eN_{D}}{2\epsilon_{0}\epsilon_{SC}}\left(\frac{R^{2}}{2}-\frac{R_{1}^{2}}{2}-R_{1}^{2}ln\left(\frac{R}{R_{1}}\right)\right)$$

The above equation is transcendental in $R_{1}$, meaning that it is not possible to find an explicit expression for $R_{1}$ as a function of $\Delta \phi_{SC}$.

(11) $$\Delta \phi_{H}=\frac{eN_{D}}{2\epsilon_{0}\epsilon_{H}}\left(R^{2}-R_{1}^{2}\right)ln\left(\frac{R_{H}}{R}\right)$$

In analogy to the approximation used in [45] for the spherical case, an approximate analytic expression is employed for the potential drop in the Gouy–Chapman layer (see Supplementary Materials for a derivation):

(12) $$\Delta \phi_{el}=\frac{4kT}{e}arctanh\left(C+\sqrt{1+C^{2}}\right)$$

where

(13) $$C=\frac{\epsilon_{el}}{\epsilon_{SC}}\frac{kT}{e}\frac{1+2BR_{H}}{R}\frac{1}{\frac{\partial \phi_{SC}}{\partial r}\left(R\right)}$$

and

(14) $$B=e\sqrt{\frac{2c_{0}}{kT\epsilon_{0}\epsilon_{el}}}.$$

For convenience, we again report here the explicit expressions for $\frac{\partial \phi_{SC}}{\partial r}\left(R\right)$:

(15) $$\frac{\partial \phi_{SC}}{\partial r}\left(R\right)=-\frac{eN_{D}}{2\epsilon_{0}\epsilon_{SC}}\left(R-\frac{R_{1}^{2}}{R}\right).$$

These equations are valid up the situation where the space charge layer fills the whole semiconductor. In this limiting case, the maximal potential drop in the semiconductor is reached:

(16) $$\Delta \phi_{SC}^{limit-cyl}=\phi_{SC}\left(R_{1}\right)-\phi_{SC}\left(R\right)=\frac{eN_{D}R^{2}}{4\epsilon_{0}\epsilon_{SC}}$$

or

(17) $$\Delta \phi_{SC}^{limit-cyl}=\phi_{SC}\left(R_{1}\right)-\phi_{SC}\left(R\right)=\frac{3R^{2}}{2A_{0}}.$$

### 2.3. Calculation Parameters

The parameters needed for the calculation are the dielectric constants $\epsilon_{SC}$, $\epsilon_{H}$, and $\epsilon_{el}$ for space charge layer, Helmholtz layer, and Gouy–Chapman layer, respectively, along with the doping density in the semiconductor $N_{D}$, the ion concentration in the electrolyte $c_{0}$, the width of the Helmholtz layer $L_{H}$, and the flatband potential $V_{fb}$. Most of these are taken directly from the experimental literature. For hematite, the value $\epsilon_{SC}$ = 57 from [46] has been used; moreover, as discussed in [11], $\epsilon_{el}$ = 64.42 has been used [11,47]. To illustrate the effect of doping in hematite, we highlight two extreme situations, one with low doping and one with high doping, for which the data have been taken from the experiments by Iandolo et al. [48] and Le Formal et al. [8], respectively. Silicon is normally present as a substitutional dopant in the 4+ oxidation state, which should be compared with the 3+ oxidation state of iron in hematite. This results in one extra electron per silicon atom. Because oxygen in the oxide lattice is in the 2− oxidation state, the oxygen vacancy usually results in two extra electrons in the material. Both cases result in n-doping being present in the oxide. The experimental data are summarised in Table 1. On the contrary, $L_{H}$ and $\epsilon_{H}$ are not available from the experimental literature; the values of $L_{H}$ = 4.4 Å and $\epsilon_{H}$ = 25.3 have been taken from theoretical works [11,35]. A thorough discussion on these theoretical values can be found in [11]. For titania, the value $\epsilon_{SC}$ = 50 has been used, as in [49]. For titania, we chose experiments with the same NaOH-based electrolyte as in the case of hematite to ensure that we could use the same dielectric constants. NaOH-based electrolytes are very common in this kind of experiment [13]. For the Helmholtz layer of titania, we used the value $L_{H}$ = 10 Å, as in [50].

## 3. Results and Discussion

**Figure 2:**
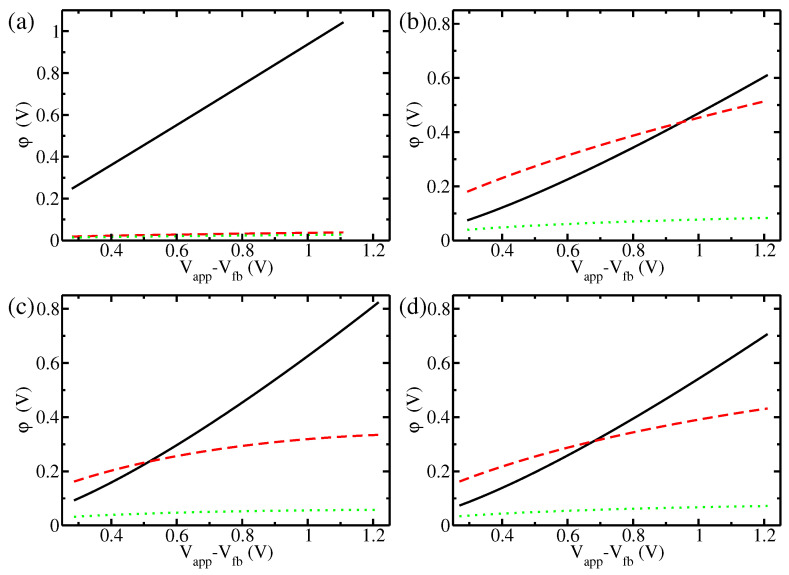
Potential drops in the various parts of the interface for hematite: potential of the space charge layer $\Delta \phi_{SC}$ (black solid line); potential of the Helmholtz layer $\Delta \phi_{H}$ (red dashed line); and potential of the Gouy–Chapman layer $\Delta \phi_{el}$ (green dotted line). The four subgraphs refer to four different situations: (**a**) planar interface at low doping, $N_{D}$ = $2.28\times 10^{18}$ ${\mathrm{cm}}^{-3}$ (as in [48]); (**b**) planar interface at high doping, $N_{D}$ = $7.00\times 10^{20}$ ${\mathrm{cm}}^{-3}$ (as in [8]); (**c**) spherical nanoparticle (radius R = 5 nm) at high doping, $N_{D}$ = $7.00\times 10^{20}$ ${\mathrm{cm}}^{-3}$ (as in [8]); (**d**) cylindrical nanorod (radius R = 5 nm) at high doping, $N_{D}$ = $7.00\times 10^{20}$ ${\mathrm{cm}}^{-3}$ (as in [8]).

For the cases of hematite with low and high doping concentrations, the potential drops in the three regions of the interface (the space charge layer, Helmholtz layer, and Gouy–Chapman layer) are shown in Figure 2 for the spherical and cylindrical geometries, with the results for the planar interface from [11] shown for comparison. In all cases, the low-doping regime displays the typical behaviour of a semiconductor interface, with by far the largest contribution to the potential drop provided by the space charge layer. On the other side, the highly doped semiconductor displays intermediate behaviour between a pure semiconductor and a metal, with a substantial portion of the potential drop being located in the Helmholtz layer. This behaviour is more pronounced in the planar interface. Counterintuitively, it is damped in the nanostructures; the reason for this is that the small radius of curvature enhances more the electric field in the space charge layer, resulting in a smaller electric field in the Helmholtz layer. In the case of high doping, the electric field in the Helmholtz layer reaches values higher than 100 mV/Å for the planar interface, while in the nanoparticle with a radius of 5 nanometers it reaches values of at most ∼80 mV/Å. Notably, 5 nm was the radius of the typical nanostructures in recent experiments with hematite [52] and titania [51]. We note that such high electric fields have been shown to lead to freezing of pure water [42]. It would be interesting to understand how the dynamics of interfacial water are affected under these conditions; however, this is beyond the scope of the present paper. On the contrary, the potential drop across the Gouy–Chapman layer, though more pronounced in the highly doped case, remains smaller than 100 mV under all considered conditions.

**Figure 3:**
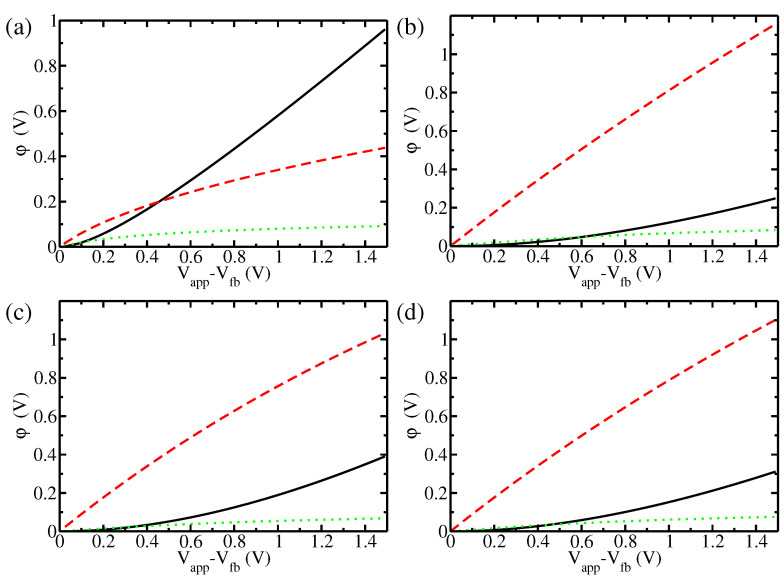
Potential drops in the various parts of the interface for titania: potential of the space charge layer $\Delta \phi_{SC}$ (black solid line); potential of the Helmholtz layer $\Delta \phi_{H}$ (red dashed line); potential of the Gouy–Chapman layer $\Delta \phi_{el}$ (green dotted line). The four subgraphs refer to four different situations: (**a**) planar interface at high doping, $N_{D}$ = $7.05\times 10^{19}$ ${\mathrm{cm}}^{-3}$ (as in [51]); (**b**) planar interface at extremely high doping, $N_{D}$ = $1.9\times 10^{21}$ ${\mathrm{cm}}^{-3}$ (as in [49]); (**c**) spherical nanoparticle (radius R = 5 nm) at extremely high doping, $N_{D}$ = $1.9\times 10^{21}$ ${\mathrm{cm}}^{-3}$ (as in [49]); (**d**) cylindrical nanorod (radius R = 5 nm) at extremely high doping, $N_{D}$ = $1.9\times 10^{21}$ ${\mathrm{cm}}^{-3}$ (as in [49]).

The trends observed in hematite are even more pronounced in titania (Figure 3) due to the even higher doping concentration reported in [49]. In this case, $N_{D}$ is so high that most of the potential drop takes place in the Helmholtz layer. Even at the highest applied potentials, $\Delta \phi_{SC}$ remains smaller than 400 meV. This behaviour is more similar to that of a metal than that of a typical semiconductor. We note that such high doping concentrations have been reported for several nanostructured oxides; for example, Cristino et al. reported $N_{D}$ = 7.5 $\times \;10^{20}$ ${\mathrm{cm}}^{-3}$ in nanostructured ${\mathrm{WO}}_{3}$ films [53], and Shaddad et al. reported $N_{D}$ = 9.25 $\times \;10^{19}$ ${\mathrm{cm}}^{-3}$ in dendritic nanostructured ${\mathrm{Bi}}_{2}$$O_{3}$ films [54].

It could be questioned whether the employed model is still valid under such high electric fields. In fact, the high electric fields predicted here for the Helmholtz layer should have a non-negligible effect on the water dynamics in the layer, probably contributing to polarisation of any water there. This in turn should affect the dielectric constant of the Helmholtz layer, decreasing its value and leading to modified behaviour on the part of the interface. While the effects of a field-dependent dielectric constant are not included in the model, it can be speculated that a reduction of the dielectric constant would further increase the electric field. However, a full treatment is beyond the scope of this paper.

At this point, it might be useful to underline the conceptual difference between the quantities considered here and the quantities obtained in typical atomistic simulations. The potentials and fields reported here are only those due to the charging of the interface away from the conditions of flatband potential. At the flatband potential, all fields and potential differences considered in the present work are zero. This is a striking difference from the typical case of atomistic simulations, where the atomic nuclei carry a point charge. There, electric fields, which we might call intrinsic atomic electric fields, are always present, and can be sizable or even larger than those reported here. However, they are always present, even at the conditions of flatband potential, where excited charges do not produce macroscopic drift currents. Therefore, the photocurrents that are of relevance for photocatalysis are related to the “macroscopic” electric fields considered in the present work, which are superimposed upon the intrinsic atomic electric fields. This explains why, for example, Sang et al. found huge electric fields (up to 3 V/Ångstrom) even in the neutral interface by means of classical molecular dynamics [50]. The fields calculated here are superimposed upon these intrinsic contributions. Thus, the situation described here is more akin to atomistic simulations in which an external electric field is imposed. Indeed, Futera et al. [55] found that water dissociates at the interface with hematite in external electric fields of 75 mV/Å and above, with strong reorganisation. However, Futera et al. also noticed that external fields of 100 mV/Å created numerical problems for their molecular dynamics algorithm. Given that in their simulations the external field was naturally screened by water polarisation, the total electric field is bound to be smaller than our value of 100 mV/Å. Thus, the high-field regime depicted here is in fact largely unexplored.

A second result of this work is the calculation of the limiting potential drop that can be sustained inside the nanostructure. This can be readily obtained from Equation (9) for a spherical nanoparticle and from Equation (17) for the case of a nanorod. Full depletion of the semiconductor corresponds to setting $R_{1}$ to zero. For the spherical nanoparticle, we obtain

(18) $$\Delta \phi_{SC}^{limit-sph}=\frac{eN_{D}R^{2}}{6\epsilon_{0}\epsilon_{SC}}=\frac{R^{2}}{A_{0}},$$

while for the nanorod we obtain

(19) $$\Delta \phi_{SC}^{limit-cyl}=\frac{eN_{D}R^{2}}{4\epsilon_{0}\epsilon_{SC}}=\frac{3R^{2}}{2A_{0}},$$

where

(20) $$A_{0}=\frac{6\epsilon_{0}\epsilon_{SC}}{eN_{D}}.$$

**Figure 4:**
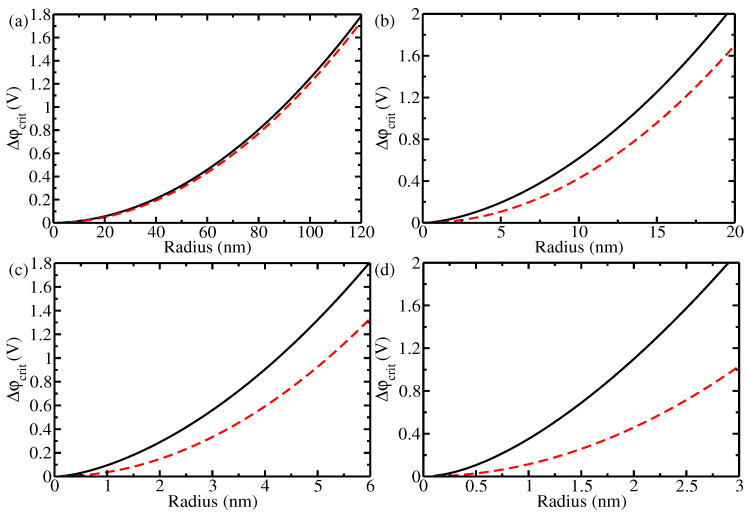
Limiting potentials $\Delta \phi_{tot}^{limit}$ = $V_{app}$− $V_{flatband}$ (black solid line) and $\Delta \phi_{SC}^{limit}$ (red dashed line) for a spherical nanoparticle, for which the space charge layer includes the whole nanoparticle in dependence of its radius. (**a**,**c**) refer to hematite: (**a**) $N_{D}$ = $2.28\times 10^{18}$ ${\mathrm{cm}}^{-3}$ (as in [48]); (**c**) $N_{D}$ = $7.00\times 10^{20}$ ${\mathrm{cm}}^{-3}$ (as in [8]). Notice the two different scales on the x-axis. (**b**,**d**) refer to titania: (**b**) $N_{D}$ = $7.05\times 10^{19}$ ${\mathrm{cm}}^{-3}$ (as in [51]); (**d**) $N_{D}$ = $1.9\times 10^{21}$ ${\mathrm{cm}}^{-3}$ (as in [49]). Notice the two different scales on the x-axis.

Figure 4 shows the limiting potential drop $\Delta \phi_{tot}^{limit}$ = $V_{app}$− $V_{flatband}$ across the interface, for which the space charge layer extends over the whole nanoparticle in dependence on the particle’s radius, as well as the corresponding potential drop $\Delta \phi_{SC}^{limit}$ across the space charge layer. In the case of low doping these two quantities almost coincide, whereas the Helmholtz contribution is substantial in the case of high doping. At low doping (Figure 4a), the space charge layer is very extended, to easily over hundreds of nanometers, leading to charge depletion even in fairly large nanostructures. On the contrary, high applied potentials are necessary at high doping (Figure 4b–d) in order to deplete nanoparticles of a few nanometers. In either case, the solid electrode has no possibility to accommodate further potential increases above the limiting potential, which are taken care of either by an increase in the electric fields in the liquid electrolyte or by charging of the substrate. This makes more precise the observation in [11] that both experiments eventually reached a regime in which the semiconductor (a thin film in [48] and a cauliflower-like structure in [8]) was completely depleted, in other words, where the space charge layer extended over the whole semiconductor.

**Figure 5:**
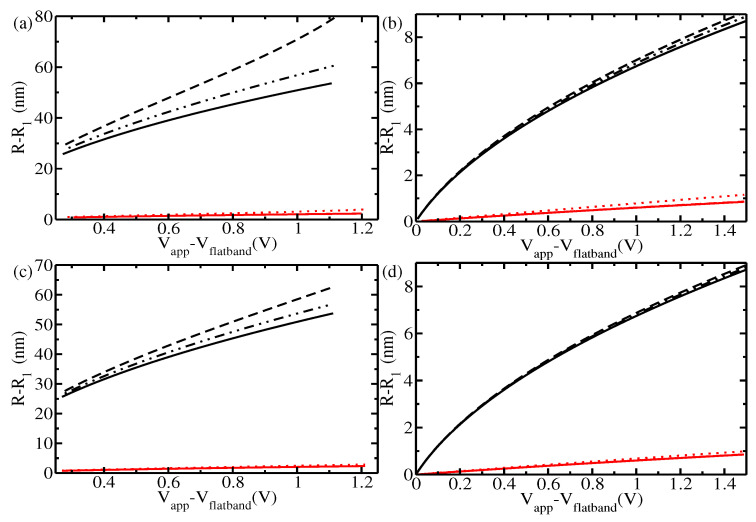
Width of the space charge layer (R-$R_{1}$) (in nm) as a function of the overall potential drop across the interface $\phi_{tot}$ (in V) for (**a**) spherical particles of hematite, (**b**) spherical particles of titania, (**c**) cylindrical structures of hematite, and (**d**) cylindrical structures of titania. (**a**,**c**) refer to hematite: radius of 500 μm in the situation of the experiment by Iandolo et al. [48] (black solid line); radius of 200 nm in the situation of the experiment by Iandolo et al. [48] (black dashed-double dotted line); radius of 100 nm in the situation of the experiment by Iandolo et al. [48] (black dashed line); radius of 500 μm in the situation of the experiment by Le Formal et al. [8] (red solid line); radius of 100 nm in the situation of the experiment by Le Formal et al. [8] (red dashed line, not visible due to overlapping with the red solid line); radius of 5 nm in the situation of the experiment by Le Formal et al. [8] (red dotted line). For the experiment by Iandolo et al., the curve with a radius of 5 nm is not shown, as at this doping density nanostructures of 5 nm are completely depleted at potentials of only a few mV. (**b**,**d**) refer to titania: radius of 500 μm in the situation of the experiment by Hernandez et al. [51] (black solid line); radius of 200 nm in the situation of the experiment by Hernandez et al. [51] (black dashed-double dotted line); radius of 100 nm in the situation of the experiment by Hernandez et al. [51] (black dashed line); radius of 500 μm in the situation of the experiment by Shaddad et al. [49] (red solid line); radius of 100 nm in the situation of the experiment by Shaddad et al. [49] (red dashed line, not visible due to overlapping with the red solid line); radius of 5 nm in the situation of the experiment by Shaddad et al. [49] (red dotted line). For the experiment by Hernandez et al., the curve with a radius of 5 nm is not shown, as at this doping density nanostructures of 5 nm are completely depleted at potentials of 200 mV (nanoparticles) or 300 mV (nanorods).

**Figure 6:**
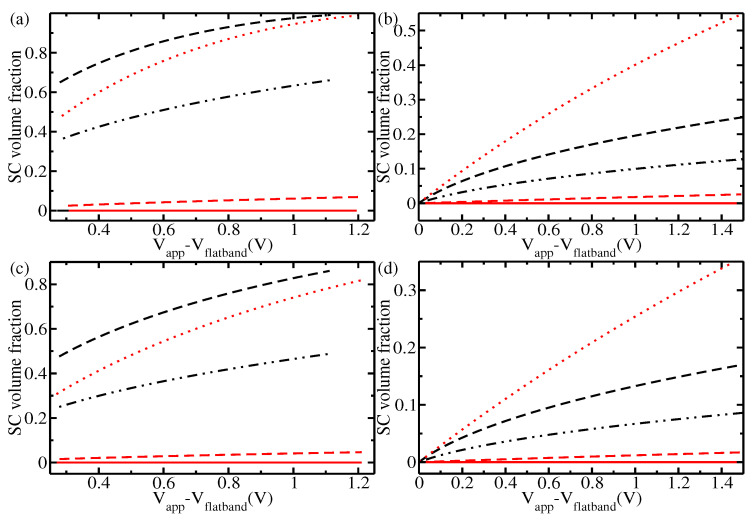
Fraction of the semiconductor taken up by the space charge layer as a function of the overall potential drop across the interface $\phi_{tot}$ (in V) for (**a**) spherical particles of hematite, (**b**) spherical particles of titania, (**c**) cylindrical structures of hematite, and (**d**) cylindrical structures of titania. (**a**,**c**) refer to hematite: radius of 500 μm in the situation of the experiment by Iandolo et al. [48] (black solid line, not visible due to overlapping with the red solid line); radius of 200 nm in the situation of the experiment by Iandolo et al. [48] (black dashed-double dotted line); radius of 100 nm in the situation of the experiment by Iandolo et al. [48] (black dashed line); radius of 500 μm in the situation of the experiment by Le Formal et al. [8] (red solid line); radius of 100 nm in the situation of the experiment by Le Formal et al. [8] (red dashed line); radius of 5 nm in the situation of the experiment by Le Formal et al. [8] (red dotted line). For the experiment by Iandolo et al., the curve with a radius of 5 nm is not shown, as at this doping density nanostructures of 5 nm are completely depleted at potentials of only a few mV. (**b**,**d**) refer to titania: radius of 500 μm in the situation of the experiment by Hernandez et al. [51] (black solid line, not visible due to overlapping with the red solid line); radius of 200 nm in the situation of the experiment by Hernandez et al. [51] (black dashed-double dotted line); radius of 100 nm in the situation of the experiment by Hernandez et al. [51] (black dashed line); radius of 500 μm in the situation of the experiment by Shaddad et al. [49] (red solid line); radius of 100 nm in the situation of the experiment by Shaddad et al. [49] (red dashed line); radius of 5 nm in the situation of the experiment by Shaddad et al. [49] (red dotted line). For the experiment by Hernandez et al., the curve with a radius of 5 nm is not shown, as at this doping density nanostructures of 5 nm are completely depleted at potentials of 200 mV (nanoparticles) or 300 mV (nanorods).

The results for the nanostructures illustrate that despite the very different doping in the two experiments, both result in situations where a large part of the semiconductor belongs to the space charge layer thanks to the fine nanostructure of the highly doped sample. To quantify this, we calculated the width and volume fraction of the space charge layer over the total volume of the semiconductor, with the results shown in Figure 5 and Figure 6, respectively. Figure 5a shows the width of the space charge layer in the spherical case as a function of the total potential drop across the interface. As expected, the width is much smaller in the case with high doping than in the case with low doping. However, as shown in Figure 6, nanostructuring of a highly doped electrode can lead to a situation where the fraction of the total volume that is taken up by the space charge layer is comparable to that in the case with low doping. The figure shows that at a doping concentration of $N_{D}$ = $7.00\times 10^{20}$ ${\mathrm{cm}}^{-3}$, a particle of 5 nm radius has a similar volume fraction of space charge layer to a particle of 100 nm at a lower doping concentration of $N_{D}$ = $2.28\times 10^{18}$ ${\mathrm{cm}}^{-3}$. Considering that it is often assumed that the holes that can reach the surface and participate in the photocatalysis are mostly produced in the space charge layer, and as such are partly protected from recombination, these results quantitatively shown how high doping concentrations can work when accompanied by nanostructuring. The width and volume fraction of the space charge layer in the cylindrical case are reported in Figure 5c,d and Figure 6c,d, respectively. Not unexpectedly, the cylindrical case is intermediate between the spherical and planar cases. Nonetheless, in this case nanostructuring again brings the volume fraction of small-radius nanorods in the highly doped case into line with that of the low-doping case for nanorods with a larger radius. Therefore, the effect of decreased space layer width can again be obviated through nanostructuring.

The electric field in the space charge layer is crucial to ensuring electron–hole separation and avoiding recombination. Therefore, it is usually assumed that an extended space charge layer leads to higher performance, as it leads to decreased recombination over a large portion of the semiconductor. This would speak in favour of low doping in the semiconductor. On the other hand, higher doping fosters conductivity inside the semiconductor, though at the price of higher recombination. Here, we show that high doping with nanostructuring makes the best of both situations, as is it possible to have a high volume fraction of the semiconductor in the space charge layer (thereby avoiding recombination) while also harvesting the advantages of high doping. Therefore, we propose that photocatalytic performance can best benefit from high doping when carried out together with fine nanostructuring of the material. In addition, we show that this could contribute to better charge transport, avoiding the pitfalls of high recombination, and provide a quantitative framework for evaluating this connection. Finally, we propose that the optimal doping concentration should increase with decreasing size of the nanostructure, a prediction that could be tested experimentally in future research.

## 4. Conclusions

In this paper, the effect of doping and nanostructuring on the electrochemical interface between an oxide (hematite or titania) and a water electrolyte has been investigated using a Poisson–Boltzmann model, including the space charge layer, Helmholtz layer, and Gouy–Chapman layer. Thanks to robust yet simple approximations for the diffuse layer, compact analytic expressions are reported for the potential drop across the interface in the cases of spherical and cylindrical nanostructures. For spherical nanoparticles and nanorods, simple formulae for the limiting potentials at which the space charge layer includes the whole semiconductor are provided. These can be used in future experiments to estimate whether full depletion of the semiconductor has been reached. At low doping, nanostructures can already be fully depleted at potentials of only a few mV.

At high doping densities, a substantial part of the potential drop is located in the Helmholtz layer, contrary to what happens in a conventional semiconductor. This results in huge electric fields in the liquid, which can be as high as 100 mV/Å, comparable to electric fields that induce freezing in pure water. This effect is slightly reduced by nanostructuring; for nanoparticles of 5 nm, the electric field in the Helmholtz layer reaches values of at most ∼80 mV/Å. The potential drop in the Gouy–Chapman layer is negligible at low doping, while at high doping it is smaller than 100 mV under all considered conditions. It is highly necessary that local electric fields and water dynamics at the interface be tested experimentally in future research.

It is usually assumed that high doping improves the charge dynamics in hematite but reduces the space charge layer width, i.e., it reduces the portion of the semiconductor from which charges can be collected. However, nanostructuring corrects the latter negative effect. We show quantitatively that in highly doped nanostructures the space charge layer can occupy a similar volume fraction as in low-doped microparticles. These results imply that the optimal doping concentration should become larger as the size of the nanostructures decreases, a behaviour that would be interesting to test experimentally.

This work provides a systematic quantitative framework for understanding the effect of doping and nanostructuring on electrochemical interfaces by providing a complete set of equations for the description of these interfaces at arbitrary doping concentrations. These results suggest that it remains necessary to better characterize the dynamics of the interface at the atomistic level, especially in the presence of high electric fields. Moreover, in future experiments it will be necessary to characterise the properties of the electrochemical interface in detail in terms of local electric fields in order to allow for direct comparison with our model.

## Figures and Tables

**Table 1:** Experimental data used in this work: doping density $N_{D}$ (${\mathrm{cm}}^{-3}$), flat band potential $V_{fb}$, onset potential in the dark, onset potential under illumination, and $c_{0}$ ion density in the electrolyte.

Reference	Dopant Type	$N_{D}$ (${\mathrm{cm}}^{-3}$)	$V_{\mathrm{fb}}$	$V_{\mathrm{onset}}^{\mathrm{dark}}$	$V_{\mathrm{onset}}^{\mathrm{illumination}}$	$c_{0}$ (M)
Hematite						
Iandolo et al. [48]	Oxygen vacancies	$2.28\times 10^{18}$	0.73	1.65	1.28	0.1056
Le Formal et al. [8]	Si	$7.00\times 10^{20}$	0.53	1.58	1.0	1.0330
Titania						
Hernandez et al. [51]	Oxygen vacancies	$7.05\times 10^{19}$	0.2	1.75	0.17	0.1
Shaddad et al. [49]	Oxygen vacancies	$1.9\times 10^{21}$	0.11	-	0.13	1.0

## Data Availability

Data is contained within the article or Supplementary Materials.

## Supplementary Materials

The following supporting information can be downloaded at: https://www.mdpi.com/article/10.3390/ma17143460/s1, Figure S1: relative error of the cylindrical approximation; Table S1: experimental data.
