# Supplementary material for: Quantitative Analysis of the Synergy of Doping and Nanostructuring of Oxide Photocatalysts
Source: Materials (Basel). 2024 Jul 12;17(14):3460. doi: 10.3390/ma17143460 (PMC11278242; doi:10.3390/ma17143460)
Supplement: Supplementary file 1 [file materials-17-03460-s001.zip › materials-3101284-supplementary.pdf]

Supporting Information: Quantitative analysis of the synergy of doping and nanostructuring of oxide photocatalysts

Nicola Seriani,[*,1] Paola Delcompare-Rodriguez,[2] Dhanshree Pandey,[1] Abhishek Kumar Adak,[1] Vikram Mahamiya,[1] Carlos Pinilla,[3] and Hala J. El-Khozondar[4]

1*The Abdus Salam International Centre for Theoretical Physics, Strada Costiera 11, 34151 Trieste, Italy*

2*Istituto Officina dei Materiali, Consiglio Nazionale delle Ricerche (CNR-IOM), Via Bonomea 265, Trieste 34136, Italy*

3*Departamento de Fisica y Geociencias, Universidad del Norte, Km 5, Via Puerto Colombia, Barranquilla 080020, Colombia*

4*Electrical Engineering and Smart Systems Department, Faculty of Engineering, Islamic University of Gaza, Gaza P.O. Box 108, Palestine*

Correspondence: nseriani@ictp.it

Poisson-Boltzmann equations in the spherical case

Here we consider the case where the system has spherical symmetry. Apart from this, all usual approximations are employed, in particular the depletion approximation. The solution for the space charge layer is from the book by Bisquert,[1] while the approximate solution for the Gouy-Chapman layer is from Ref.[2] The semiconductor is a sphere. The model assumes that the center of the sphere is electrically connected to the outer circuit. The semiconductor is a sphere of radius R, and the space charge layer is located in the region between a radius R_1 and R ($0 \leq R_1 \leq R$).

Space charge layer

Using the spherical expression of the laplacian and the depletion approximation, the Poisson equations in the SC layer reads

$$\frac{\partial^2 \phi_{SC}}{\partial r^2} + \frac{2}{r}\frac{\partial \phi_{SC}}{\partial r} = -\frac{eN_D}{\epsilon_0 \epsilon_{SC}} \tag{S1}$$

subject to the boundary conditions

$$\frac{\partial \phi_{SC}}{\partial r}(R_1) = 0; \phi_{SC}(R_1) = 0 \tag{S2}$$

However, it is more convenient to use Gauss' theorem for r ($R_1 \leq$ r $\leq$ R):

$$4\pi r^2 \frac{\partial \phi_{SC}}{\partial r} = -\frac{eN_D}{\epsilon_0 \epsilon_{SC}} \frac{4\pi}{3}\left(r^3 - R_1^3\right) \tag{S3}$$

$$\frac{\partial \phi_{SC}}{\partial r} = -\frac{eN_D}{3\epsilon_0 \epsilon_{SC}}\left(r - \frac{R_1^3}{r^2}\right) \tag{S4}$$

Integration leads to

$$\phi_{SC}(r) - \phi_{SC}(R_1) = -\frac{eN_D}{3\epsilon_0 \epsilon_{SC}} \int_{R_1}^{r} dr' \left(r' - \frac{R_1^3}{r'^2}\right) = \tag{S5}$$

$$\phi_{SC}(r) - \phi_{SC}(R_1) = -\frac{eN_D}{3\epsilon_0\epsilon_{SC}}\left\{\left(\frac{r^2}{2} - \frac{R_1^2}{2} + R_1^3\left(\frac{1}{r} - \frac{1}{R_1}\right)\right\}\right. \tag{S6}$$

$$\Delta\phi_{SC} = \phi_{SC}(R_1) - \phi_{SC}(R) = \frac{eN_D}{3\epsilon_0\epsilon_{SC}}\left\{\frac{R^2 - R_1^2}{2} + R_1^3\left(\frac{1}{R} - \frac{1}{R_1}\right)\right\} \tag{S7}$$

$$\Delta\phi_{SC} = \phi_{SC}(R_1) - \phi_{SC}(R) = \frac{eN_D}{3\epsilon_0\epsilon_{SC}}\left\{\frac{R^2 - R_1^2}{2} + R_1^2\left(\frac{R_1 - R}{R}\right)\right\} \tag{S8}$$

$$\Delta\phi_{SC} = \frac{eN_D}{6\epsilon_0\epsilon_{SC}}\frac{1}{R}\left(R^3 - 3R_1^2R + 2R_1^3\right) \tag{S9}$$

This equation connects $\Delta\phi_{SC}$ with R_1, and it allows to determine the latter from the former. The equation is a cubic equation in R_1:

$$2R_1^3 - (3R)R_1^2 + R^3 - \frac{6\epsilon_0\epsilon_{SC}R}{eN_D}\Delta\phi_{SC} = 0 \tag{S10}$$

This is a cubic equation of the form:

$$ax^3 + bx^2 + cx + d = 0 \tag{S11}$$

In our case, c = 0. In the following, for brevity let's introduce the quantity

$$A_0 = \frac{6\epsilon_0\epsilon_{SC}}{eN_D} \tag{S12}$$

so that the cubic equation can be written as

$$2R_1^3 - (3R)R_1^2 + R^3 - A_0R\Delta\phi_{SC} = 0 \tag{S13}$$

Explicit solutions to the cubic equation are known since Cardano in the XVI century. The most convenient way to express them is to transform the cubic equation in the depressed cubic form

$$t^3 + pt + q = 0 \tag{S14}$$

which can be obtatined from

$$ax^3 + bx^2 + cx + d = 0 \tag{S15}$$

through the substitution x = t $-\frac{b}{3a}$. The depressed cubic equation has the three solutions in trigonometric form

$$t_k = 2\sqrt{-\frac{p}{3}}cos\left(\frac{1}{3}arccos\left(\frac{3q}{2p}\sqrt{-\frac{3}{p}}\right) - \frac{2\pi k}{3}\right), k = 0, 1, 2 \tag{S16}$$

where

$$p = \frac{3ac - b^2}{3a^2} \tag{S17}$$

$$q = \frac{2b^3 - 9abc + 27a^2d}{27a^3} \tag{S18}$$

In our case this translates into

$$p = -\frac{3}{4}R^2 \tag{S19}$$

$$q = \frac{1}{2}R^3 - \frac{1}{2}A_0R\Delta\phi_{SC} \tag{S20}$$

So the solutions read

$$t_k = Rcos\left(\frac{1}{3}arccos\left(-1 + 2\frac{A_0}{R^2}\Delta\phi_{SC}\right) - \frac{2\pi k}{3}\right), k = 0, 1, 2 \tag{S21}$$

Numerical inspection shows that there is a real solution R_1 between 0 and R for k = 1 and $0 \leq \Delta\phi_{SC} \leq \frac{R^2}{A_0}$

Important equations for the space charge layer

Let us summarise the results for the space charge layer, and the relations between potential drop, electric field (specially that at the boundary, which enters as boundary conditions also in the other sections), and width of the space charge layer.

As a function of R_1, the radius at which the space charge layer starts, electric field and

potential drops are expressed as

$$\frac{\partial \phi_{SC}}{\partial r}(r) = -\frac{eN_D}{3\epsilon_0\epsilon_{SC}}\left(r - \frac{R_1^3}{r^2}\right) \tag{S22}$$

In particular, the electric field at the interface with the electrolyte (r=R) is

$$\frac{\partial \phi_{SC}}{\partial r}(R) = -\frac{eN_D}{3\epsilon_0\epsilon_{SC}}\left(R - \frac{R_1^3}{R^2}\right) \tag{S23}$$

This expression will enter as a boundary condition in the study of the other sectors of the interface, see below. The potential drop across the space charge layer is

$$\Delta\phi_{SC} = \frac{eN_D}{6\epsilon_0\epsilon_{SC}}\frac{1}{R}\left(R^3 - 3R_1^2R + 2R_1^3\right) \tag{S24}$$

On the other side, R_1 itself is a function of the potential drop across the space charge layer. Experimentally, the system is controlled through the application of an external bias, so it is more convenient to express R_1 and the electric field as a function of the potential drop $\Delta\phi_{SC}$:

$$R_1 = \frac{R}{2} + t_1 = \frac{R}{2} + Rcos\left(\frac{1}{3}arccos\left(-1 + 2\frac{A_0}{R^2}\Delta\phi_{SC}\right)\right) \tag{S25}$$

where

$$A_0 = \frac{6\epsilon_0\epsilon_{SC}}{eN_D} \tag{S26}$$

The electric field reads

$$\frac{\partial \phi_{SC}}{\partial r}(r) = -\frac{eN_D}{3\epsilon_0\epsilon_{SC}}\left(r - \frac{R^3}{4r^2}\big(\frac{1}{2} + cos(D)(6cos(D)) + 2cos(2D) + 5)\big)\right) \tag{S27}$$

where

$$D = \frac{1}{3}arccos\left(-1 + 2\frac{A_0}{R^2}\Delta\phi_{SC}\right) - \frac{2\pi}{3} \tag{S28}$$

The electric field at the interface between semiconductor and electrolyte is then

$$\frac{\partial \phi_{SC}}{\partial r}(R) = -\frac{eN_D}{3\epsilon_0\epsilon_{SC}} R\left(\frac{7}{8} - \frac{1}{4}cos(D)\Big(6cos(D)) + 2cos(2D) + 5\Big)\right) \tag{S29}$$

This last equation enters into the boundary condition of the Helmholtz layer and represents therefore the connection between $\Delta\phi_{SC}$, $\Delta\phi_H$, and $\Delta\phi_{el}$, as shown below.

Extreme cases

Here we check that the above results lead to expected behaviour in extreme cases. For $\Delta\phi_{SC} = 0$

$$t_1 = Rcos\left(\frac{1}{3}arccos\Big(-1\Big) - \frac{2\pi}{3}\right) = \frac{R}{2} \tag{S30}$$

$$R_1 = t_1 + \frac{R}{2} = R \tag{S31}$$

This result is obvious and consistent with common knowledge: when the bands are flat, the space charge layer disappears.

At the other extreme, $\Delta\phi_{SC} = \frac{R^2}{A_0}$, one obtains

$$t_1 = Rcos\left(\frac{1}{3}arccos\Big(1\Big) - \frac{2\pi}{3}\right) = -\frac{R}{2} \tag{S32}$$

$$R_1 = t_1 + \frac{R}{2} = 0 \tag{S33}$$

Which again explains the upper limit on $\Delta\phi_{SC}$: it is the value of bending for which the space charge layer coincides with the whole sphere, and no further width increase is possible. This is the first interesting result of the calculation: for a spherical semiconducting electrode of radius R and donor concentration N_D, the space charge layer coincides with the whole electrode at $\Delta\phi_{SC} = \frac{R^2}{A_0}$, or, more explicitly

$$\Delta\phi_{SC}^{limit-sph} = \frac{eN_DR^2}{6\epsilon_0\epsilon_{SC}} \tag{S34}$$

This equation can be used to test whether in experiments this limit has been reached and overcome.

Helmholtz layer

At the interface between the semiconductor and the Helmholtz layer, the condition applies

$$\epsilon_{SC}\frac{\partial\phi_{SC}}{\partial r}(R) = \epsilon_H\frac{\partial\phi_H}{\partial r}(R) \tag{S35}$$

which means

$$\frac{\partial\phi_H}{\partial r}(R) = \frac{\epsilon_{SC}}{\epsilon_H}\frac{\partial\phi_{SC}}{\partial r}(R) \tag{S36}$$

To derive electric field and potential in the Helmholtz layer, we can apply again Gauss' theorem to conclude that

$$\frac{\partial\phi_H}{\partial r}(r) = \frac{\partial\phi_H}{\partial r}(R)\frac{R^2}{r^2} \tag{S37}$$

between R and R$_H$. Integrating this expression

$$\Delta\phi_H = -\int_R^{R_H}\frac{\partial\phi_H}{\partial r}(r)dr \tag{S38}$$

$$\Delta\phi_H = -\frac{\partial\phi_H}{\partial r}(R)R^2\left(\frac{1}{R} - \frac{1}{R_H}\right) \tag{S39}$$

$$\Delta\phi_H = -\frac{\epsilon_{SC}}{\epsilon_H}\frac{\partial\phi_{SC}}{\partial r}(R)R^2\left(\frac{1}{R} - \frac{1}{R_H}\right) \tag{S40}$$

This expression relates directly the potential drop to the boundary condition in r=R through $\frac{\partial\phi_{SC}}{\partial r}(R)$. In the following, $\Delta\phi_H$ is expressed as function of R$_1$ or as a function of $\Delta\phi_{SC}$. First, the expression for $\Delta\phi_H$ containing R$_1$ is explicitly obtained:

$$\Delta\phi_H = \frac{eN_D}{3\epsilon_0\epsilon_H}\left(R - \frac{R_1^3}{R^2}\right)R^2\left(\frac{1}{R} - \frac{1}{R_H}\right) \tag{S41}$$

or, alternatively

$$\Delta\phi_H = \frac{eN_D}{3\epsilon_0\epsilon_H}\Big(R^3 - R_1^3\Big)\Big(\frac{1}{R} - \frac{1}{R_H}\Big) \tag{S42}$$

$\Delta\phi_H$ can also be expressed as a function of $\Delta\phi_{SC}$, which is more convenient for our purposes. Starting from Eq. S40, the expression of $\frac{\partial\phi_{SC}}{\partial r}(R)$ (Eq. S29) is substituted, to obtain:

$$\Delta\phi_H = \frac{eN_D}{3\epsilon_0\epsilon_H}R^3\frac{R_H - R}{R \cdot R_H}\Big(\frac{7}{8} - \frac{1}{4}cos(D)\Big(6cos(D)) + 2cos(2D) + 5\Big)\Big) \tag{S43}$$

where

$$D = \frac{1}{3}arccos\Big(-1 + 2\frac{A_0}{R^2}\Delta\phi_{SC}\Big) - \frac{2\pi}{3} \tag{S44}$$

Planar limit for the Helmholtz layer

In the large-radius limit, it reduces to the planar case. It is more convenient to use the expression involving the radii, because there the geometric interpretation is more direct. To show what happens in the large-radius limit, we need the decomposition

$$R^3 - R_1^3 = (R - R_1)(R^2 + RR_1 + R_1^2) \tag{S45}$$

$$\Delta\phi_H = \frac{eN_D}{3\epsilon_0\epsilon_H}(R - R_1)(R^2 + RR_1 + R_1^2)\frac{R_H - R}{RR_H} \tag{S46}$$

In the limit R, R_1, $R_H \to \infty$, we have

$$lim_{R's\to\infty}\frac{R^2 + RR_1 + R_1^2}{RR_H} = 3 \tag{S47}$$

$$lim_{R's\to\infty}\Delta\phi_H = \frac{eN_D}{\epsilon_0\epsilon_H}(R - R_1)(R_H - R) \tag{S48}$$

where $R-R_1$ is the width of the space charge layer L_{SC} and R_H-R is the width of the Helmholtz layer L_H.

$$lim_{R's\to\infty}\Delta\phi_H = \frac{eN_D}{\epsilon_0\epsilon_H}L_{SC}L_H \tag{S49}$$

This corresponds to the equation for the planar case reported in Ref.,[4] taking into account their expression for L_{SC}.

Gouy-Chapman layer

Let's now turn our attention to the diffuse Gouy-Chapman layer. With the usual assumptions, the Poisson-Boltzmann equations reads

$$\frac{d^2\phi_{el}}{dr^2} + \frac{2}{r}\frac{d\phi_{el}}{dr} = \frac{ec_0}{\epsilon_0\epsilon_{el}}\left(exp\left(\frac{e(\phi_{el}(r) - \phi_{el}(+\infty))}{kT}\right) - exp\left(\frac{-e(\phi_{el}(r) - \phi_{el}(+\infty))}{kT}\right)\right) \quad \text{(S50)}$$

with the boundary conditions:

$$\epsilon_H \frac{\partial\phi_H}{\partial r}(R_H) = \epsilon_{el}\frac{\partial\phi_{el}}{\partial r}(R_H) \quad \text{(S51)}$$

$$lim_{r\to\infty}\frac{\partial\phi_{el}}{\partial r}(r) = 0 \quad \text{(S52)}$$

$$lim_{r\to\infty}\phi_{el}(r) = \phi_{el}(+\infty) \quad \text{(S53)}$$

No exact solution is known for this equation. However, Yao et al.[2] have worked out an approximate analytical expression for its solution. Basically, they propose to take the exact expression for the planar case:

$$\phi_{el}^{planar}(r) = \phi_{el}^{planar}(+\infty) + \frac{2kT}{e} ln\left(\frac{1 + Aexp\Big(-e\sqrt{\frac{2c_0}{kT\epsilon_0\epsilon_{el}}}(x - L_H)\Big)}{1 - Aexp\Big(-e\sqrt{\frac{2c_0}{kT\epsilon_0\epsilon_{el}}}(x - L_H)\Big)}\right) \quad \text{(S54)}$$

where

$$A = \frac{exp\left(\frac{e(\phi(L_H)-\phi(+\infty))}{2kT}\right) - 1}{exp\left(\frac{e(\phi(L_H)-\phi(+\infty))}{2kT}\right) + 1} \quad \text{(S55)}$$

Under depletion conditions, this means that 0≤A<1. The approximate solution they suggested is built through the substitution

$$e\sqrt{\frac{2c_0}{kT\epsilon_0\epsilon_{el}}}(x - L_H) \to e\sqrt{\frac{2c_0}{kT\epsilon_0\epsilon_{el}}}(r - R_H) + ln\left(\frac{r}{R_H}\right) \tag{S56}$$

This ensures that the low-potential limit (i.e. the linearized Poisson-Boltzmann equation) and the large-radius limit (i.e. the planar case) are satisfied, and the authors showed that the approximation is pretty robust also in the other cases.[2]

Let's write the approximate form explicitly:

$$\phi_{el}(r) = \phi_{el}(+\infty) + \frac{2kT}{e}ln\left(\frac{1 + A\frac{R_H}{r}exp\Big(-e\sqrt{\frac{2c_0}{kT\epsilon_0\epsilon_{el}}}(r - R_H)\Big)}{1 - A\frac{R_H}{r}exp\Big(-e\sqrt{\frac{2c_0}{kT\epsilon_0\epsilon_{el}}}(r - R_H)\Big)}\right) \tag{S57}$$

or

$$\phi_{el}(r) = \phi_{el}(+\infty) + \frac{4kT}{e}arctanh\left(A\frac{R_H}{r}exp\left(-e\sqrt{\frac{2c_0}{kT\epsilon_0\epsilon_{el}}}(r - R_H)\right)\right) \tag{S58}$$

To determine $\Delta\phi_{el} = \phi_{el}(+\infty) - \phi_{el}(R_H)$, we need to consider the boundary condition in R_H

$$\epsilon_{el}\frac{\partial\phi_{el}}{\partial r}(R_H) = \epsilon_H\frac{\partial\phi_H}{\partial r}(R_H) = \epsilon_H\frac{\partial\phi_H}{\partial r}(R)\frac{R^2}{R_H^2} \tag{S59}$$

$$\epsilon_{el}\frac{\partial\phi_{el}}{\partial r}(R_H) = \epsilon_H\frac{\partial\phi_H}{\partial r}(R_H) = \epsilon_{SC}\frac{\partial\phi_{SC}}{\partial r}(R)\frac{R^2}{R_H^2} \tag{S60}$$

To calculate the derivative, let's introduce the constant B for a more compact description:

$$B = e\sqrt{\frac{2c_0}{kT\epsilon_0\epsilon_{el}}} \tag{S61}$$

So that

$$\phi_{el}(r) = \phi_{el}(+\infty) + \frac{4kT}{e}arctanh\left(A\frac{R_H}{r}exp(-B(r - R_H))\right) \tag{S62}$$

Its derivative reads

$$\frac{d\phi_{el}}{dr}(r) = \frac{4kT}{e}\left(\frac{1}{1 - A^2\frac{R_H^2}{r^2}exp(-2B(r-R_H))}\right)R_H A\left\{-\frac{1}{r^2}exp(-B(r-R_H))+\frac{1}{r}exp(-B(r-R_H))(-B)\right\} = \tag{S63}$$

$$= \frac{4kT}{e}\left(\frac{1}{1 - A^2\frac{R_H^2}{r^2}exp(-2B(r-R_H))}\right)A\frac{R_H}{r}exp(-B(r-R_H))\left\{-\frac{1}{r} - B\right\} \tag{S64}$$

Simplifying and calculating it in R$_H$

$$\frac{d\phi_{el}}{dr}(R_H) = \frac{4kT}{e}\frac{A}{1-A^2}\left\{-\frac{1+BR_H}{R_H}\right\} \tag{S65}$$

This expression can be inserted in the boundary condition at R$_H$:

$$\epsilon_{el}\frac{4kT}{e}\frac{A}{1-A^2}\left\{-\frac{1+BR_H}{R_H}\right\} = \epsilon_H\frac{\partial\phi_H}{\partial r}(R_H) \tag{S66}$$

Here, the r-dependence of ϕ_H and the boundary condition at R can be used to connect $\frac{d\phi_{el}}{dr}(R_H)$ with the functions in the space charge layer:

$$\epsilon_{el}\frac{4kT}{e}\frac{A}{1-A^2}\left\{-\frac{1+BR_H}{R_H}\right\} = \epsilon_H\frac{\partial\phi_H}{\partial r}(R)\frac{R^2}{R_H^2} \tag{S67}$$

$$\epsilon_{el}\frac{4kT}{e}\frac{A}{1-A^2}\left\{-\frac{1+BR_H}{R_H}\right\} = \epsilon_{SC}\frac{\partial\phi_{SC}}{\partial r}(R)\frac{R^2}{R_H^2} \tag{S68}$$

Since we have explicit expressions for $\frac{\partial\phi_{SC}}{\partial r}(R)$, this equation connects ϕ_{el} with either R$_1$ or directly $\Delta\phi_{SC}$. We need to solve for A:

$$\epsilon_{SC}\frac{\partial\phi_{SC}}{\partial r}(R)\frac{R^2}{R_H^2}A^2 - \epsilon_{el}\frac{4kT}{e}\left\{-\frac{1+BR_H}{R_H}\right\}A - \epsilon_{SC}\frac{\partial\phi_{SC}}{\partial r}(R)\frac{R^2}{R_H^2} = 0 \tag{S69}$$

$$\frac{\partial\phi_{SC}}{\partial r}(R)A^2 - \frac{\epsilon_{el}}{\epsilon_{SC}}\frac{4kT}{e}\frac{R_H}{R^2}(1+BR_H)A - \frac{\partial\phi_{SC}}{\partial r}(R) = 0 \tag{S70}$$

which yields

$$_1A_2 = \frac{1}{2}\frac{\epsilon_{el}}{\epsilon_{SC}}\frac{4kT}{e}\frac{R_H}{R^2}(1+BR_H)\frac{1}{\frac{\partial \phi_{SC}}{\partial r}(R)} \pm \frac{\sqrt{(\frac{\epsilon_{el}}{\epsilon_{SC}})^2(\frac{4kT}{e})^2\frac{R_H^2}{R^4}(1+BR_H)^2 + 4(\frac{\partial \phi_{SC}}{\partial r}(R))^2}}{2\frac{\partial \phi_{SC}}{\partial r}(R)} \tag{S71}$$

$$_1A_2 = \frac{1}{2}\frac{\epsilon_{el}}{\epsilon_{SC}}\frac{4kT}{e}\frac{R_H}{R^2}(1+BR_H)\frac{1}{\frac{\partial \phi_{SC}}{\partial r}(R)} \pm \sqrt{1+\frac{1}{4}(\frac{\epsilon_{el}}{\epsilon_{SC}})^2(\frac{4kT}{e})^2\frac{R_H^2}{R^4}(1+BR_H)^2(\frac{1}{\frac{\partial \phi_{SC}}{\partial r}(R)})^2} \tag{S72}$$

Remember that A is defined as

$$A = \frac{exp\left(\frac{e(\phi_{el}(R_H)-\phi_{el}(+\infty))}{2kT}\right)-1}{exp\left(\frac{e(\phi(R_H)-\phi(+\infty))}{2kT}\right)+1} = tanh\left(\frac{e(\phi(R_H)-\phi(+\infty))}{4kT}\right) \tag{S73}$$

In order to satisfy the $-1 \leq \mathrm{A} \leq 1$, remembering that we are considering situations where $\frac{\partial \phi_{SC}}{\partial r}(R) < 0$, we must take the solution with $+$. This yields:

$$\phi_{el}(R_H) - \phi_{el}(+\infty) = \frac{4kT}{e}arctanh\left(C+\sqrt{1+C^2}\right) \tag{S74}$$

where

$$C = \frac{1}{2}\frac{\epsilon_{el}}{\epsilon_{SC}}\frac{4kT}{e}\frac{R_H}{R^2}(1+BR_H)\frac{1}{\frac{\partial \phi_{SC}}{\partial r}(R)} \tag{S75}$$

and

$$B = e\sqrt{\frac{2c_0}{kT\epsilon_0\epsilon_{el}}} \tag{S76}$$

For convenience, we report here again the explicit expressions for $\frac{\partial \phi_{SC}}{\partial r}(R)$:

$$\frac{\partial \phi_{SC}}{\partial r}(R) = -\frac{eN_D}{3\epsilon_0\epsilon_{SC}}\left(R - \frac{R_1^3}{R^2}\right) \tag{S77}$$

or

$$\frac{\partial \phi_{SC}}{\partial r}(R) = -\frac{eN_D}{3\epsilon_0\epsilon_{SC}}R\left(\frac{7}{8} - \frac{1}{4}cos(D)\left(6cos(D)\right) + 2cos(2D) + 5\right)\right) \tag{S78}$$

where

$$D = \frac{1}{3} arccos\bigg(-1 + 2\frac{A_0}{R^2}\Delta\phi_{SC}\bigg) - \frac{2\pi}{3} \tag{S79}$$

and

$$A_0 = \frac{6\epsilon_0\epsilon_{SC}}{eN_D} \tag{S80}$$

This provides the connection with the independent variable $\Delta\phi_{SC}$.

Poisson-Boltzmann equations in the cylindrical case

The cylindrical case is also very interesting in view of the existence of nanostructures with cylindrical symmetry, such as nanotubes and nanorods. In this case, we assume that the most internal part of the cylindrical structure is the metallic lead to the outer circuit, followed by the semiconductor, and the electrolyte is external. We use a similar notation to the previous section: the semiconductor is a cylinder of radius R, and the space charge layer is located in the region between a radius R_1 and R ($0 \leq R_1 \leq R$). R is a geometric feature of the nanostructure, while R_1 depends on the applied bias. The general boundary conditions are $\phi_{SC}(R_1) = 0$, $\frac{\partial\phi_{SC}}{\partial r}(R_1) = 0$, $lim_{r->\infty}\phi_{el}(r) = \phi_{el}(+\infty)$.

Space charge layer

Using the cylindrical expression of the laplacian and the depletion approximation, the Poisson equations in the SC layer reads

$$\frac{\partial^2\phi_{SC}}{\partial r^2} + \frac{1}{r}\frac{\partial\phi_{SC}}{\partial r} = -\frac{eN_D}{\epsilon_0\epsilon_{SC}} \tag{S81}$$

subject to the boundary conditions

$$\frac{\partial\phi_{SC}}{\partial r}(R_1) = 0; \phi_{SC}(R_1) = 0 \tag{S82}$$

Here it is equally easy to use Gauss' theorem or to integrate directly. Let's integrate directly:

$$\frac{1}{r}\frac{\partial}{\partial r}\left(r\frac{\partial \phi_{SC}}{\partial r}\right) = -\frac{eN_D}{\epsilon_0 \epsilon_{SC}} \tag{S83}$$

$$\frac{\partial}{\partial r}\left(r\frac{\partial \phi_{SC}}{\partial r}\right) = -\frac{eN_D}{\epsilon_0 \epsilon_{SC}} r \tag{S84}$$

Integrating both sides between R_1 and r

$$\frac{\partial}{\partial r}\left(r\frac{\partial \phi_{SC}}{\partial r}\right) = -\frac{eN_D}{\epsilon_0 \epsilon_{SC}} r \tag{S85}$$

$$r\frac{\partial \phi_{SC}}{\partial r} = -\frac{eN_D}{\epsilon_0 \epsilon_{SC}}\left(\frac{r^2}{2} - \frac{R_1^2}{2}\right) \tag{S86}$$

$$\frac{\partial \phi_{SC}}{\partial r} = -\frac{eN_D}{2\epsilon_0 \epsilon_{SC}}\left(r - \frac{R_1^2}{r}\right) \tag{S87}$$

Integrating again between R_1 and r

$$\phi_{SC}(r) = -\frac{eN_D}{2\epsilon_0 \epsilon_{SC}}\left(\frac{r^2}{2} - \frac{R_1^2}{2} - R_1^2 ln(\frac{r}{R_1})\right) \tag{S88}$$

which leads to the relation between $\Delta\phi_{SC}$ and R_1:

$$\Delta\phi_{SC} = \phi_{SC}(R_1) - \phi_{SC}(R) = \frac{eN_D}{2\epsilon_0 \epsilon_{SC}}\left(\frac{R^2}{2} - \frac{R_1^2}{2} - R_1^2 ln(\frac{R}{R_1})\right) \tag{S89}$$

The equation is trascendent in R_1 and it is therefore not possible to find an explicit expression for R_1 as a function of $\Delta\phi_{SC}$. As in the previous section, we need also the electric field in R because this connects the field in the space charge layer with that in the Helmholtz and Gouy-Chapman layers:

$$\frac{\partial \phi_{SC}}{\partial r}(R) = -\frac{eN_D}{2\epsilon_0 \epsilon_{SC}}\left(R - \frac{R_1^2}{R}\right) \tag{S90}$$

Given that, in this case, it is not possible to find an explicit expression for R_1 as a function of $\Delta\phi_{SC}$, in the following we are going to express all quantities as functions of R_1, and will

report numerical results for the dependence on $\Delta\phi_{SC}$.

Critical potential

Eq. S89 can be used to identify the critical potential $\Delta\phi_{SC}^{limit-cyl}$ at which the space charge layer coincides with the whole semiconductor. Taking the limit $R_1 \rightarrow 0$ in Eq. S89 one obtains:

$$\Delta\phi_{SC}^{limit-cyl} = \phi_{SC}(R_1) - \phi_{SC}(R) = \frac{eN_D R^2}{4\epsilon_0\epsilon_{SC}} \tag{S91}$$

or

$$\Delta\phi_{SC}^{limit-cyl} = \phi_{SC}(R_1) - \phi_{SC}(R) = \frac{3R^2}{2A_0} \tag{S92}$$

Helmholtz layer

At the interface between the semiconductor and the Helmholtz layer, the condition applies

$$\epsilon_{SC}\frac{\partial\phi_{SC}}{\partial r}(R) = \epsilon_H\frac{\partial\phi_H}{\partial r}(R) \tag{S93}$$

which means

$$\frac{\partial\phi_H}{\partial r}(R) = \frac{\epsilon_{SC}}{\epsilon_H}\frac{\partial\phi_{SC}}{\partial r}(R) \tag{S94}$$

To derive electric field and potential in the Helmholtz layer, we can apply Gauss' theorem to conclude that

$$\frac{\partial\phi_H}{\partial r}(r) = \frac{\partial\phi_H}{\partial r}(R)\frac{R}{r} \tag{S95}$$

between R and R_H. Integrating this expression

$$\Delta\phi_H = \int_{R_H}^{R}\frac{\partial\phi_H}{\partial r}(r)dr = \int_{R_H}^{R}\frac{\partial\phi_H}{\partial r}(R)\frac{R}{r}dr \tag{S96}$$

$$\Delta\phi_H = \frac{\partial\phi_H}{\partial r}(R)Rln(\frac{R}{R_H}) \tag{S97}$$

Using the boundary condition and the expression for $\frac{\partial \phi_{SC}}{\partial r}(R)$

$$\Delta\phi_H = \frac{eN_D}{2\epsilon_0\epsilon_H}\Big(R^2 - R_1^2\Big)ln(\frac{R_H}{R}) \tag{S98}$$

which connects $\Delta\phi_H$ and R_1.

Gouy-Chapman layer

Let's now turn our attention to the diffuse Gouy-Chapman layer. With the usual assumptions, the Poisson-Boltzmann equations reads

$$\frac{d^2\phi_{el}}{dr^2} + \frac{1}{r}\frac{d\phi_{el}}{dr} = \frac{ec_0}{\epsilon_0\epsilon_{el}}\Bigg(exp\Bigg(\frac{e\Big(\phi_{el}(r) - \phi_{el}(+\infty)\Big)}{kT}\Bigg) - exp\Bigg(\frac{-e\Big(\phi_{el}(r) - \phi_{el}(+\infty)\Big)}{kT}\Bigg)\Bigg) \tag{S99}$$

with the boundary conditions:

$$\epsilon_H\frac{\partial\phi_H}{\partial r}(R_H) = \epsilon_{el}\frac{\partial\phi_{el}}{\partial r}(R_H) \tag{S100}$$

$$lim_{r\to\infty}\frac{\partial\phi_{el}}{\partial r}(r) = 0 \tag{S101}$$

Just as in the spherical case, no exact solution is known. Analogously to that case,[2] an approximate solution can be obtained from the one for the planar case through an appropriate substitution, which reads

$$e\sqrt{\frac{2c_0}{kT\epsilon_0\epsilon_{el}}}(x - L_H) \to e\sqrt{\frac{2c_0}{kT\epsilon_0\epsilon_{el}}}(r - R_H) + \frac{1}{2}ln\Bigg(\frac{r}{R_H}\Bigg) \tag{S102}$$

This substitution adds the term $\frac{1}{2}ln\Big(\frac{r}{R_H}\Big)$, which is intermediate between 0 (planar case) and $ln\Big(\frac{r}{R_H}\Big)$ (speherical case). Then, the exact solution for the planar case, which is

$$\phi_{el}^{planar}(r) = \phi_{el}^{planar}(+\infty) + \frac{2kT}{e}ln\Bigg(\frac{1 + Aexp\Big(-e\sqrt{\frac{2c_0}{kT\epsilon_0\epsilon_{el}}}(x - L_H)\Big)}{1 - Aexp\Big(-e\sqrt{\frac{2c_0}{kT\epsilon_0\epsilon_{el}}}(x - L_H)\Big)}\Bigg) \tag{S103}$$

where

$$A = \frac{exp\left(\frac{e(\phi(L_H)-\phi(+\infty))}{2kT}\right)-1}{exp\left(\frac{e(\phi(L_H)-\phi(+\infty))}{2kT}\right)+1} \tag{S104}$$

becomes

$$\phi_{el}(r) = \phi_{el}(+\infty) + \frac{2kT}{e} ln\left(\frac{1 + A\sqrt{\frac{R_H}{r}} exp\Big(-e\sqrt{\frac{2c_0}{kT\epsilon_0\epsilon_{el}}}(r-R_H)\Big)}{1 - A\sqrt{\frac{R_H}{r}} exp\Big(-e\sqrt{\frac{2c_0}{kT\epsilon_0\epsilon_{el}}}(r-R_H)\Big)}\right) \tag{S105}$$

or

$$\phi_{el}(r) = \phi_{el}(+\infty) + \frac{4kT}{e} arctanh\left(A\sqrt{\frac{R_H}{r}} exp\left(-e\sqrt{\frac{2c_0}{kT\epsilon_0\epsilon_{el}}}(r-R_H)\right)\right) \tag{S106}$$

To determine $\Delta\phi_{el} = \phi_{el}(R_H) - \phi_{el}(+\infty)$, we need to consider the boundary condition in R$_H$

$$\epsilon_{el}\frac{\partial\phi_{el}}{\partial r}(R_H) = \epsilon_H\frac{\partial\phi_H}{\partial r}(R_H) = \epsilon_H\frac{\partial\phi_H}{\partial r}(R)\frac{R}{R_H} \tag{S107}$$

$$\epsilon_{el}\frac{\partial\phi_{el}}{\partial r}(R_H) = \epsilon_H\frac{\partial\phi_H}{\partial r}(R_H) = \epsilon_{SC}\frac{\partial\phi_{SC}}{\partial r}(R)\frac{R}{R_H} \tag{S108}$$

To calculate the derivative, let's introduce the constant B for a more compact description:

$$B = e\sqrt{\frac{2c_0}{kT\epsilon_0\epsilon_{el}}} \tag{S109}$$

So that

$$\phi_{el}(r) = \phi_{el}(+\infty) + \frac{4kT}{e} arctanh\left(A\sqrt{\frac{R_H}{r}} exp(-B(r-R_H))\right) \tag{S110}$$

Its derivative reads

$$\frac{d\phi_{el}}{dr}(r) = -\frac{4kT}{e}\left(\frac{1}{1 - A^2\frac{R_H}{r} exp(-2B(r-R_H))}\right)\sqrt{\frac{R_H}{r}} Aexp(-B(r-R_H))\left\{\frac{1}{2r} + B\right\} \tag{S111}$$

We now calculate the derivative in $r = R_H$ and impose the boundary condition

$$\epsilon_{el}\frac{\partial\phi_{el}}{\partial r}(R_H) = \epsilon_{SC}\frac{\partial\phi_{SC}}{\partial r}(R)\frac{R}{R_H} \tag{S112}$$

resulting in

$$-\frac{2kT}{e}\frac{A}{1-A^2}\left\{\frac{1+2BR_H}{R_H}\right\} = \frac{\epsilon_{SC}}{\epsilon_{el}}\frac{R}{R_H}\frac{\partial\phi_{SC}}{\partial r}(R) \tag{S113}$$

$$-\frac{2kT}{e}A\left\{\frac{1+2BR_H}{R}\right\}\frac{\epsilon_{el}}{\epsilon_{SC}}\frac{1}{\frac{\partial\phi_{SC}}{\partial r}(R)} = 1-A^2 \tag{S114}$$

Let's rearrange term to bring this quadratic equation in A in standard form:

$$A^2 - \frac{2kT}{e}\frac{1+2BR_H}{R}\frac{\epsilon_{el}}{\epsilon_{SC}}\frac{1}{\frac{\partial\phi_{SC}}{\partial r}(R)}A - 1 = 0 \tag{S115}$$

which yields the solutions

$${}_1A_2 = \frac{\epsilon_{el}}{\epsilon_{SC}}\frac{kT}{e}\frac{1+2BR_H}{R}\frac{1}{\frac{\partial\phi_{SC}}{\partial r}(R)} \pm \sqrt{(\frac{\epsilon_{el}}{\epsilon_{SC}})^2(\frac{kT}{e})^2(\frac{1+2BR_H}{R})^2\frac{1}{(\frac{\partial\phi_{SC}}{\partial r}(R))^2}+1} \tag{S116}$$

$${}_1A_2 = \frac{\epsilon_{el}}{\epsilon_{SC}}\frac{kT}{e}\frac{1+2BR_H}{R}\frac{1}{\frac{\partial\phi_{SC}}{\partial r}(R)} \pm \sqrt{1+(\frac{\epsilon_{el}}{\epsilon_{SC}})^2(\frac{kT}{e})^2\frac{1+2BR_H}{R}(\frac{1}{\frac{\partial\phi_{SC}}{\partial r}(R)})^2} \tag{S117}$$

Remember that A is defined as

$$A = \frac{exp\left(\frac{e(\phi_{el}(R_H)-\phi_{el}(+\infty))}{2kT}\right)-1}{exp\left(\frac{e(\phi(R_H)-\phi(+\infty))}{2kT}\right)+1} = tanh\left(\frac{e(\phi(R_H)-\phi(+\infty))}{4kT}\right) \tag{S118}$$

In order to satisfy the $-1 \leq A \leq 1$, remembering that we are considering situations where $\frac{\partial\phi_{SC}}{\partial r}(R) < 0$, we must take the solution with +. This yields:

$$\phi_{el}(R_H) - \phi_{el}(+\infty) = \frac{4kT}{e}arctanh\left(C + \sqrt{1+C^2}\right) \tag{S119}$$

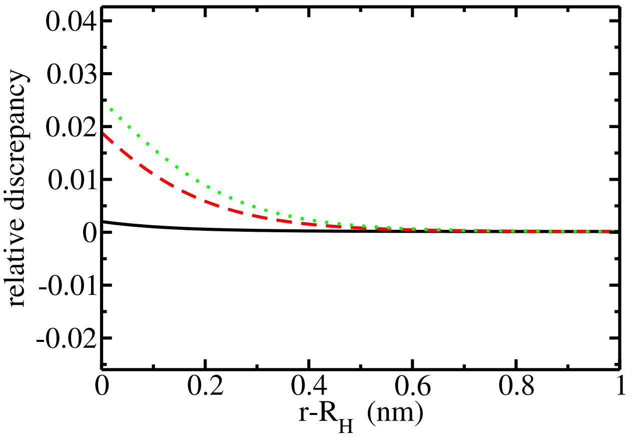

Figure S1: Relative error of the approximation for the Gouy-Chapman layer in the cylindrical approximation. Calling A the lefthand side of the Poisson-Boltzmann equation for the cylindrical case, Eq. S99, and B the righthand side, both calculated for the approximate solution of Eq. S99, the reported relative error is (A-B)/B. As shown, the relative error is never larger than 3% and decays quickly with the distance from R_H.

where

$$C = \frac{\epsilon_{el}}{\epsilon_{SC}} \frac{kT}{e} \frac{1 + 2BR_H}{R} \frac{1}{\frac{\partial \phi_{SC}}{\partial r}(R)} \tag{S120}$$

and

$$B = e\sqrt{\frac{2c_0}{kT\epsilon_0\epsilon_{el}}} \tag{S121}$$

For convenience, we report here again the explicit expressions for $\frac{\partial \phi_{SC}}{\partial r}(R)$:

$$\frac{\partial \phi_{SC}}{\partial r}(R) = -\frac{eN_D}{2\epsilon_0\epsilon_{SC}}\left(R - \frac{R_1^2}{R}\right) \tag{S122}$$

In this case it is not possible to find an explicit expression of $\frac{\partial \phi_{SC}}{\partial r}(R)$ as function of $\Delta\phi_{SC}$.

To show that the approximation is legitimate, we show that the approximate solution satifies the Poisson-Boltzmann equation to a good approximation. The expressions on the lefthand (A) and righthand (B) side of the equation have been evaluated for the approximate form with parameters taken from the experiment by LeFormal et al.,[3] and the relative error (A-B)/B is reported in Fig. S1. The discrepancy is never larger than 3%, and decays very quickly with the distance from R_H, thereby validating the approximation.

Table S1: Geometry, dopant type, dopant density (N_D), width of space charge layer (L_{SC}), flat band potential (V_{fb}), and ion concentration at the electrolyte (C_0) for different oxide photocatalyst. R: radius of nanorod, RHE: reversible hydrogen electrode, SCE: Saturated Calomel Electrode, SHE: Standard Hydrogen Electrode (Pt)

Sl no.	Geometry	Dopant type	N_D (cm^{-3})	L_{SC} (nm)	V_{fb} (V)	C_0 (M)	Reference electrode
			**Hematite (α-Fe_2O_3)**				
1	Nanorod (R=25nm)	Pristine	7.5×10^{18}	22	0.12	1.0 M NaOH	RHE[5]
		Mn	1.0×10^{20}	6	0.50		
2	Nanorod	Pristine	2.66×10^{17}		-0.55	1.0 M NaOH	Ag/AgCl[6]
		5% Mn	3.02×10^{19}		-0.52		
3	Nanorod	Sn	$0.48-2.43 \times 10^{20}$		0.50−0.45	1.0 M NaOH	Ag/AgCl[7]
4	Nanorod	Ru	9.7×10^{20}		0.52		RHE[8]
5	Nanotube	Ni	2.39×10^{20}		-0.60	1.0 M KOH	Ag/AgCl[9]
6	Nanowires	P	1.01×10^{20}			1.0 M NaOH	RHE[10]
7	Nanowires/Nano corals	Sn	$1.89-5.38 \times 10^{19}$		0.47−0.49	1.0 M NaOH	RHE[11]
8	Thin film	10% Al	1.30×10^{22}		-0.74	1.0 M NaOH	Ag/AgCl[12]
9	Thin film	Zr	2.60×10^{21}	71	-0.74	1.0 M NaOH	SCE[13]
10	Wormlike	Pt	2.77×10^{18}		0.5−0.6	1.0 M NaOH	Ag/AgCl[14]
			**Titania (TiO_2)**				
11	Nanowire	Pristine	3.86×10^{18}		-0.89	1.0 M KOH	Ag/AgCl[15]
		W-doped	2.06×10^{18}		-0.87		
		Etched	1.36×10^{18}		-0.58		
		W-doped/etched	5.04×10^{18}		-0.60		
12	Nanorod	Pristine	8.75×10^{17}	137	-0.15	1.0 M NaOH	Ag/AgCl[16]
		Fe-doped (5 mM)	0.69×10^{17}	398	0.15		
		Fe-doped (7.5 mM)	6.90×10^{17}	167	-0.30		
		Fe-doped (10 mM)	6.54×10^{17}	172	-0.30		
13	Nanorod	Pristine	5.60×10^{17}		-0.53	3.0 M KCl	Pt coated[17] FTO
		1La-doped	1.39×10^{18}		-0.61		
		4La-doped	8.75×10^{18}		-0.86		
14	Nanorod arrays	Pristine	4.5×10^{17}	99	0.20	0.5 M $NaClO_4$	Ag/AgCl[18]
15	Nanoparticle	Pristine	5.0×10^{17}		-0.60	0.05 M KCl	Ag/AgCl[19]
	Nanowire		2.0×10^{18}		-1.11		
16	Nanotubes	Pristine	7.05×10^{19}		0.20	0.1 M NaOH	RHE[20]
			**Other transition metal oxides**				
17	WO_3 films Nanostructured	Colloidal	1.3×10^{20}	30-40	0.21	1.0 M H_2SO_4	SCE[21]
		Anodic	7.5×10^{20}	5-6	0.15	1.0 M H_2SO_4	
		Colloidal	1.1×10^{20}	30−40	0.15	0.1 M $TBAHSO_4$	
		Anodic	7.1×10^{20}	5−6	0.11	0.1 M $TBAHSO_4$	
		Colloidal	0.9×10^{20}	30−40	0.16	0.1 M $LiHSO_4$	
		Anodic	7.4×10^{20}	5-6	0.11	0.1 M $LiHSO_4$	
18	Nanostructured	Pristine	8.82×10^{19}		0.40	0.1 M Phosphate buffer solution	RHE[22]
	Bi_2O_3 films	Oxygen vacancy	9.25×10^{19}		0.37		

References

(1) J. Bisquert, Nanostructured energy devices, Equilibrium Concepts and Kinetics, CRC Press, Universitat Jaume I, Castelló, Spain, 2015.

(2) Z. Yao, M. J. Bowick, and X. Ma, J. Chem. Phys. 136, 044106 (2012).

(3) F. Le Formal, N. Tetreault, M. Cornuz, T. Moehl, M. Grä tzel, and K. Sivula, Chem. Sci. 2, 737 (2011).

(4) P. A. Delcompare-Rodriguez, and N. Seriani, J. Chem. Phys. 155, 114701 (2021).

(5) Gurudayal, L. M. Peter, L. H. Wong, and F. F. Abdi, ACS Appl. Mater. Interfaces 9, 41265−41272 (2017).

(6) Gurudayal, S. Y. Chiam, M. H. Kumar, P. S. Bassi, H. L. Seng, J. Barber, and L. H. Wong, ACS Appl. Mater. Interfaces. 6, 5852−5859 (2014).

(7) Gurudayal, R. A. John, P. P. Boix, C. Yi, C. Shi, M. C. Scott, S. A. Veldhuis, A. M. Minor, S. M. Zakeeruddin, L. H. Wong, M. Grätzel and N. Mathews, ChemSusChem 10, 2449−2456 (2017).

(8) X. Guo, L. Wang, and Y. Tan, Nano Energy 16, 320−328 (2015).

(9) W. Cheng, J. He, Z. Sun, Y. Peng, T. Yao, Q. Liu, Y. Jiang, F. Hu, Z. Xie, B. He, and S. Wei, J. Phys. Chem. C 116, 24060−24067 (2012).

(10) Y. Zhang, S. Jiang, W. Song, P. Zhou, H. Ji, W. Ma, W. Hao, C. Chen, and J. Zhao, Energy Environ. Sci. 8, 1231 (2015).

(11) Y. Ling, G. Wang, D. A. Wheeler, J. Z. Zhang, and Y. Li, Nano Lett. 11, 2119−2125 (2011).

(12) A. Kleiman-Shwarsctein, M. N. Huda, A. Walsh, Y. Yan, G. D. Stucky, Y. Hu, M. M. Al-Jassim, and E. W. McFarland, Chem. Mater. 22, 510−517 (2010).

(13) P. Kumar, P. Sharma, R. Shrivastav, S. Dass, and V. R. Satsangi, Int. J. Hydrogen Energy, 36, 2777−2784 (2011).

(14) J. Y. Kim, G. Magesh, D. H. Youn, J. W. Jang, J. Kubota, K. Domen, and J. S. Lee, Sci. Rep. 3, 2681 (2013).

(15) Y. Wang, Y. Zhang, J. Tang, H. Wu, M. Xu, Z. Peng, X. Gong, and G. Zheng, ACS Nano 7, 9375−9383 (2013).

(16) W. Chakhari, J. B. Naceur, S. B. Taieb, I. B. Assaker, and R. Chtourou, J. Alloys Compd. 708, 862−870 (2017).

(17) S. Sadhuab, and P. Poddar, RSC Advances, 3, 10363 (2013).

(18) A. Wolcott, W. A. Smith, T. R. Kuykendall, Y. Zhao, and J. Z. Zhang, Small, 5, 104–111 (2009).

(19) G. Wang, Q. Wang, W. Lu, and J. Li, J. Phys. Chem. B, 110, 22029−22034 (2006).

(20) S. Hernandez, D. Hidalgo, A. Sacco, A. Chiodoni, A. Lamberti, V. Cauda, E. Tressoa, and G. Saracco, Phys. Chem. Chem. Phys., 17, 7775−7786 (2015).

(21) V. Cristino, S. Marinello, A. Molinari, S. Caramori, S. Carli, R. Boaretto, R. Argazzi, L. Meda, and C. A. Bignozzi, J. Mater. Chem. A, 4, 2995−3006 (2016).

(22) M. N. Shaddad, P. Arunachalam, M. Hezam, N. M. AL-Saeedan, S. Gimenez, J. Bisquert, and A. M. Al-Mayouf, Int. J. Hydrogen Energy. 46, 23702−23714 (2021).
